# Monitoring of brain oxygen saturation (INVOS) in a protocol to direct blood transfusions during cardiac surgery: a prospective randomized clinical trial

**DOI:** 10.1186/1749-8090-8-145

**Published:** 2013-06-07

**Authors:** George Vretzakis, Stavroula Georgopoulou, Konstantinos Stamoulis, Vassilios Tassoudis, Dimitrios Mikroulis, Athanasios Giannoukas, Nikolaos Tsilimingas, Menelaos Karanikolas

**Affiliations:** 1 Cardiac Anesthesia Unit, Anesthesiology Clinic, University of Thessaly, Biopolis, Larissa, Greece; 2 Department of Cardiac Surgery, Democritus University, Alexandroupolis, Greece; 3 Vascular Department, University of Thessaly, Biopolis, Larissa, Greece; 4 Cardiac Surgery Clinic, University of Thessaly, Biopolis, Larissa, Greece; 5 Department of Anesthesiology, Washington University School of Medicine, St. Louis, Missouri, 63110, USA

**Keywords:** INVOS, Cardiac surgery, Anesthesia, Transfusion, Fluid restriction, Near-infrared spectroscopy

## Abstract

**Background:**

Blood transfusions are common in cardiac surgery, but have been associated with increased morbidity and long-term mortality. Efforts to reduce blood product use during cardiac surgery include fluid restriction to minimize hemodilution, and protocols to guide transfusion decisions. INVOS is a modality that monitors brain tissue oxygen saturation, and could be useful in guiding decisions to transfuse. However, the role of INVOS (brain tissue oxygen saturation) as part of an algorithm to direct blood transfusions during cardiac surgery has not been evaluated. This study was conducted to investigate the value of INVOS as part of a protocol for blood transfusions during cardiac surgery.

**Methods:**

Prospective, randomized, blinded clinical trial, on 150 (75 per group) elective cardiac surgery patients. The study was approved by the Institution Ethics committee and all patients gave written informed consent. Data were initially analyzed based on “intention to treat”, but subsequently were also analyzed “per protocol”.

**Results:**

When protocol was strictly followed (“per protocol analysis”), compared to the control group, significantly fewer patients monitored with INVOS received any blood transfusions (46 of 70 patients in INVOS group vs. 55 of 67 patients in the control group, p = 0.029). Similarly, patients monitored with INVOS received significantly fewer units of red blood cell transfusions intraoperatively (0.20 ± 0.50 vs. 0.52 ± 0.88, p = 0.008) and overall during hospital stay (1.31 ± 1.20 vs. 1.82 ± 1.46, p = 0.024). When data from all patients (including patient with protocol violation) were analyzed together (“intention to treat analysis”), the observed reduction of blood transfusions in the INVOS group was still significant (51 of 75 patients transfused in the INVOS group vs. 63 of 75 patients transfused in the control group, p = 0.021), but the overall number of units transfused per patient did not differ significantly between the groups (1.55 ± 1.97 vs. 1.84 ± 1.41, p = 0.288).

**Conclusions:**

Our data suggest that INVOS could be a useful tool as part of an algorithm to guide decisions for blood transfusion in cardiac surgery. Additional data from rigorous, well designed studies are needed to further evaluate the role of INVOS in guiding blood transfusions in cardiac surgery, and circumvent the limitations of this study.

**Trial registration:**

ClinicalTrials.gov: NCT00879463

## Background

Cardiac surgery is associated with significant use of blood products. Although anemia must be avoided in these operations [1,2], data suggest that blood transfusions are associated with worse outcomes [3,4], and restrictive transfusion practices (hemoglobin concentration maintained between 7.0 and 9.0 g/dL) reduce organ dysfunction and cost, without adversely affecting outcome [5,6]. A recent randomized controlled trial (RCT) in cardiac surgery showed that a restrictive transfusion strategy targeting hematocrit 24% reduced RBC use without increasing complications or mortality [7]. Furthermore, the number of transfused RBC units was a risk factor for worse outcomes and mortality in regression analysis.

Several variables associated with total red cell mass, such as preoperative anemia, female gender and small body size, are predictors of transfusion in cardiac surgery [8-10]. Hematocrit and hemoglobin values are markedly affected by positive fluid balance while on CPB without true loss of erythrocytes. Existing guidelines underline the importance of limiting hemodilution [11,12].

Blood transfusions based only on hematocrit value seem unjustified in cardiac surgery. The lack of a transfusion “threshold” and data from studies evaluating restrictive blood transfusion strategies suggest that hematocrit alone does not optimally support decisions to transfuse.

Monitoring of brain tissue oxygen saturation with near-infrared spectroscopy (INVOS) is a method for evaluating the balance of brain oxygen supply vs. consumption. Published data suggest that INVOS values have positive correlation with arterial hemoglobin oxygen saturation, arterial pCO2 values and hemoglobin (or hematocrit) values and negative correlation with patient age [13,14]. The effect of anesthetic depth on INVOS values has not been thoroughly evaluated, but data from a small RCT have shown higher INVOS values with deeper levels of sevoflurane or desflurane anesthesia [15].

Our experience while conducting earlier studies and in everyday clinical practice suggests that brain oxygen saturation with near-infrared spectroscopy (INVOS) monito ring may help limit RBC use during CPB. The present study was conducted to investigate the hypothesis that use of intra-operative INVOS monitoring can reduce blood transfusions in patients undergoing cardiac surgery under CPB with a restrictive parenteral fluid protocol and blood salvage techniques.

## Methods

### Patient selection and management

This prospective study was conducted in a tertiary care University Hospital over a 16-month period, after approval from the Institution Ethics committee. Written informed consent was obtained from all patients before entering the study. The study was registered at the http://www.clinicaltrials.gov registration site, (registration number NCT00879463).

Inclusion criteria were elective cardiac surgery under CPB, with no age or ASA physical status classification limit. Exclusion criteria were emergency or re-do operations, combined cardiac - carotid surgery and operations with minimal extracorporeal flow (surgery of the ascending aorta) or circulatory arrest. Patients with hematologic disease (including anemia requiring preoperative blood product transfusion), coagulation abnormality, advanced cirrhosis and renal dysfunction (creatinine >50% upper limit of normal value) were excluded. Antiplatelet medications (except aspirin) were discontinued at least 72 hours before surgery. Acute normovolemic hemodilution, retrograde autologous priming of the CPB circuit and pharmacologic agents to decrease blood loss were not used in any patient.

Patients were randomly assigned to one of two groups: Group A with intraoperative INVOS monitoring and Group B without INVOS (control). Group assignment originated from a sequentially numbered sealed envelope containing a randomization code. All patients received standardized anesthesia and intraoperative care, and were operated by the same team (surgeon, assistants and perfusionist) under standardized conditions with intra-operative cell salvage. All personnel (including the surgical team, perfusionist, nursing and ICU personnel) involved in the care of these patients were blinded to group assignment. Similarly, all investigators who collected data were also blinded. However, the anesthesiologist in charge of each case had access to the INVOS data and, obviously, was not blinded.

All patients received total intravenous anesthesia with propofol, remifentanil and cis-atracurium. Monitoring included mixed venous oximetry plus continuous cardiac output recording (Oximetry TD catheter, Edwards Lifesciences, Germany) and bispectral index (BIS/XP, Aspect Medical Systems, USA). Near-infrared spectroscopy was used to monitor cerebro-vascular hemoglobin oxygen saturation with the INVOS 5100 device (Somanetics, USA) in patients assigned to group A. Anticoagulation was achieved with heparin 300 IU/kg and Activated Clotting Time (ACT) > 400 s was required before initiating Cardiopulmonary Bypass (CPB). The CPB pump and tubing (Stockert SIII, Germany; circuit: Custom Pack, Dideco, Italy) were primed with 1400–2000 mls of crystalloid, based on patient somatometric characteristics. Pump flow was 2.3-2.5 liter/min/m^2^. All patients received antegrade blood cardioplegia. Isolated coronary artery bypass graft (CABG) patients were operated under mild passive hypothermia down to 33.5-34.0°C, while systemic drift to 32.0°C was applied on all other patients. Active rewarming to 37.5°C bladder temperature and proper cardiac reperfusion were applied on all patients. After weaning from CPB, heparin was neutralized by protamine 3 mg/kg, and the remaining CPB circuit blood was washed, centrifuged (Electa, Dideco, Italy) and re-transfused. Red cell salvage continued until the chest was closed.

All patients, regardless of group allocation, were treated using a restrictive parenteral fluid administration protocol, as described in our previous study [16]. Hemodynamic instability was managed using an algorithm aimed at limiting unnecessary fluid administration. If corrective measures based on the algorithm were not adequate, the anesthesiologist was free to act according to his/her judgment.

All patients were admitted to the ICU postoperatively, and received the same hypnotic-analgesic regimen (propofol and remifentanil). INVOS monitoring was not used in the ICU. Criteria for weaning from mechanical ventilation included hemodynamic stability with minimal or no catecholamine support, absence of significant dysrhythmias, absence of major bleeding, core body temperature > 36°C, proper level of consciousness and acceptable arterial blood gas values with good respiratory mechanics. Postoperative pain was controlled with intravenous morphine infusion. Patients were transferred to the ward when their clinical condition and laboratory findings were acceptable.

### Perioperative RBC transfusion

Perioperative transfusion decisions were made by the attending anesthesiologist. In group A (INVOS), decisions were as follows: If mean INVOS value from both hemispheres was less than 60 regardless of baseline values, (criterion a) or INVOS decreased by 20% or more compared to the mean value during pulmonary artery catheter insertion (criterion b), the patient was candidate for transfusion, but was transfused only if hematocrit from the arterial blood-gas analysis was indicating the need for transfusion (see below: indications in group B). Patients with low hematocrit values who did not meet the INVOS criteria (a or b, as described above) did not receive blood transfusions.

In group B (control group, no INVOS) transfusion decisions were based on hematocrit-based rules as follows: During aortic cross-clamp, allogeneic blood was not given if hematocrit was >21%. For values ≤17%, one unit of RBC was transfused. When hematocrit was between 17-21%, anesthesiologists could decide based on their clinical judgment. After aortic clamp removal and before weaning from CPB (usually near the completion of the last proximal anastomosis or during cardiac reperfusion), RBCs were transfused for hematocrit less than 21%. After weaning from CPB and re-transfusion of salvaged blood, patients were transfused for hematocrit ≤24%.

In the postoperative period, fluid and blood transfusion therapy was directed by the ICU staff who were blinded to patient group assignment and were not part of the investigator team. Consequently, while in the ICU, patients were transfused for hematocrit ≤24%, while transfusion decisions for hematocrit values between 24-30% were taken after evaluation in a multimodal manner, regardless of group allocation.

### Data collection and statistical analysis

Sample size calculation was based on the following assumptions: 75% of patients expected to be transfused in the control group, with a reduction of this percentage to 50% being considered a meaningful improvement, a = 0.05 and power 0.8. Under these assumptions, the required sample size for chi-square analysis is 57 patients per group. However, we decided to enroll up to 75 patients per group, in order to allow for potential problems, such as patient attrition, missing data or protocol violations.

Hematocrit values were recorded in both groups at the following time points: preoperatively, after arterial line placement, after anesthesia induction, 10 minutes after initiation of CPB, before termination of CPB, at the end of surgery, 6 and 12 hours after admission to the ICU and at the time of discharge from the hospital. For each patient, IV fluids (including drug infusions and fluids used for flushing of lines) and urine output before CPB, during CPB and from the end of CPB until the end of surgery were recorded. The amount of priming and cardioplegia solutions, additional fluid given during CPB, hemofiltration and pump residual volumes were also recorded. Then, based on these numbers, fluid balances were calculated for the period before CPB, during CPB and for the entire procedure. In addition, INVOS values were recorded for all patients assigned to group A.

While designing the study, we decided to define a cut-off point for separating normal vs. prolonged length of hospital stay. Data from our cardiac surgery department showed that patients undergoing these types of procedures without complications had hospital stay of 9 days or less. Therefore, in order to minimize subjectivity when recording complications, we defined complications as events that required some specific acute medical therapy or intervention resulting in prolonged (>9 days) hospital stay or death.

Based on our earlier experience, protocol violations were pre-defined as: A) Significant postoperative hemorrhage leading to transfusion of more than 8 units of RBCs. B) Intraoperative disengagement from the RBC transfusion protocol based on clinical judgment. As “transfusion threshold” remains unclear for the CPB period, the attending anesthesiologist had the liberty to transfuse RBCs in patients where they were concerned that, based on medical history and/or co-morbid conditions, peri-operative anemia could lead to complications. C) Overall intraoperative fluid balance greater than +1.5 L (this value was chosen because it is greater than mean + 2SD in our previous report [16], therefore strongly indicating that the restrictive protocol was not applied). D) In group A patients, when an apparent drop of INVOS was not consistent in both hemispheres, the patient was excluded from the protocol, and the anesthesiologist had to call the trial coordinator.

Data were collected by blinded investigators and were stored in a secure electronic database. All statistical analysis was conducted using the SPSS 15.0 for Windows statistical software package (SPSS Inc, Chicago, IL), except for chi-square analysis, which was conducted the Epi Info statistical software package, which is freely available from the CDC (Centers for Disease Control and Prevention) website, at http://wwwn.cdc.gov/epiinfo/html/downloads.htm.

For each patient, data were evaluated for completeness and reliability by the study coordinator after discharge from the hospital. Based on our hypothesis, the primary outcome of the study was the number of RBC units transfused during the operation. Collected data also included transfusions in the ICU and on the ward, ICU and hospital length of stay, morbidity and mortality, and these data were used as secondary outcomes for comparisons between the two groups. Postoperative mortality was defined as death within 30 days of discharge from the hospital. The numbers of grafts, number of transfused RBC units, nights in ICU and days until hospital discharge were analyzed as continuous variables. “Transfusion” was analyzed as a categorical variable. Normality of continuous variables was evaluated with the Kolmogorov–Smirnov test, and continuous variables were compared between the two groups using the student’s two-tailed t-test. Comparisons of categorical variables were made using chi-square test. P-values less than 0.05 were considered significant for all comparisons.

After concluding the study, data from all 150 patients were initially analyzed based on the “intention to treat” principle. Subsequently, we also conducted data analysis based on the “per protocol” principle, after identifying and excluding cases of protocol violation.

## Results

**Figure 1:**
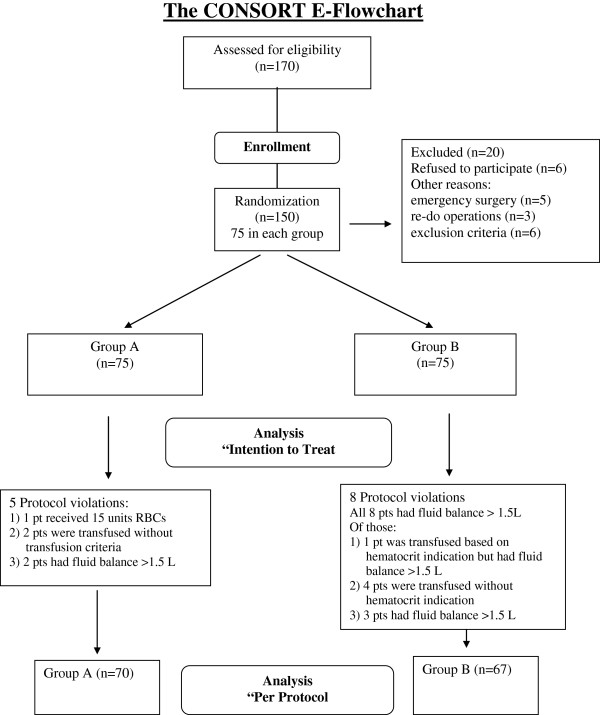
CONSORT flowchart showing the flow of patients through the study and data analysis.

In total, 150 patients were enrolled, and there were no cases of missing data. Despite occasional difficulty with placing both BIS and INVOS sensors in group A, all sensors were successfully applied and produced BIS and INVOS data of adequate quality on all group A patients. The CONSORT flowchart showing the flow of patients through the study and data analysis is presented in Figure 1. Demographic, clinical and perioperative data for both groups are summarized in Table 1, and did not differ significantly between groups. INVOS data recorded in group A are summarized in Table 2. Hematocrit values throughout the operation, during ICU stay and until the day of discharge, and data on fluid balance are shown in Table 3. Fluid balance for the entire procedure was calculated as [(total intravenous fluids + pump prime + total cardioplegia + any other “ extra ” volume in the CPB machine) − (total urine production + hemofiltration volume + saver net filtration + residual CPB circuit volume)], varied from −950 to 2550 ml in group A and from −550 to 2500 ml in group B. All variables shown on Table 3 did not differ significantly between the two groups.

**Table 1:** Demographic and clinical data by group

	**Group A**	**Group B**	**P**
	**(INVOS, n = 75)**	**(Control, n = 75)**	
Age (yrs)	67.3 ± 8.5	65.9 ± 9.5	*0*.*329*
Female n (%)	12 (16.0%)	15 (20.0%)	*0*.*523*
Weight (kg)	79.1 ± 14.4	75.9 ± 13.0	*0*.*140*
Height (cm)	166.9 ± 8.3	166.7 ± 8.9	*0*.*893*
BMI	28.4 ± 4.6	27.2 ± 3.5	*0*.*084*
BSA (m^2^)	1.86 ± 0.19	1.83 ± 0.20	*0*.*281*
LVEF (%)	47.4 ± 10.8	48.3 ± 8.0	*0*.*536*
Prior MI, n (%)	42 (56.0%)	40 (53.3%)	*0*.*742*
Diabetes, n (%)	17 (22.6%)	19 (25.3%)	*0*.*702*
Hypertension, n (%)	62 (82.6%)	59 (78.6%)	*0*.*535*
COPD, n (%)	16 (21.3%)	16 (21.3%)	*1*.*000*
Preoperative Hct (%)	39.5 ± 3.90	40.4 ± 4.53	*0*.*246*
**Operation**			
CABG (isolated), n (%)	63 (84.0%)	58 (77.3%)	*0*.*348*
Number of grafts	207 in 67 pts	192 in 66 pts	
MVR, n (%)	3 (4.0%)	3 (4.0%)	
MVR/CABG, n (%)	1 (1.3%)	0 (0.0%)	
AVR, n (%)	5 (6.6%)	4 (5.3%)	
AVR/CABG, n (%)	3 (4.0%)	8 (10.6%)	
ASD repair, n (%)	0 (0.0%)	2 (2.6%)	
CPB time (min)	88.8 ± 18.2	93.7 ± 29.8	*0*.*216*
Operation time (min)	249.9 ± 41.9	248.0 ± 59.2	*0*.*812*
**Postoperative data**			
hmv*	16.4 ± 24.7	14.6 ± 10.3	*0*.*558*
ICU LOS**	2.7 ± 3.8	2.7 ± 3.6	*0*.*999*
hospital LOS***	10.9 ± 3.6	10.2 ± 10.7	*0*.*181*
LOS > 9^◊^, n (%)	51 (68.0%)	41 (54.7%)	*0*.*132*
Complications, n (%)	14 (18.6%)	12 (16.0%)	*0*.*666*
Death, n (%)	1 (1.3%)	1 (1.3%)	*1*.*000*

* *hmv* hours of mechanical ventilation (ICU).

** ICU length of stay in nights.

*** Hospital LOS in days (not including days in hospital before surgery).

^◊^ denotes the number of patients with hospital LOS greater than 9 days.

**Table 2:** Intraoperative INVOS values (group A)

	**Before induction**		**During line insertion**		**“Worse” on CBP**	
Side	R	L	R	L	R	L
Min	47	47	38	43	36	37
Max	93	85	89	93	80	81
Mean	66.56	66.29	70.44	70.87	58.29	58.63
SD	7.31	7.48	8.65	9.28	7.89	8.22

**Table 3:** Hematocrit values, intravenous fluids and fluid balance by group

**Hematocrit values (%)**	**Group A**	**Group B**	**P**
	**(INVOS, n = 75)**	**(Control, n = 75)**	
Preoperative	39.54 ± 3.90	40.38 ± 4.53	*0*.*246*
After arterial line placement	38.45 ± 4.32	38.68 ± 4.40	*0*.*765*
After anesthesia induction	38.19 ± 4.61	37.84 ± 4.53	*0*.*655*
After first cardioplegia	20.20 ± 3.60	20.16 ± 3.83	*0*.*947*
End of CPB	23.07 ± 3.45	23.26 ± 3.03	*0*.*721*
End of operation	27.55 ± 4.18	27.50 ± 4.15	*0*.*943*
6 hours in the ICU	28.15 ± 3.38	28.79 ± 3.32	*0*.*263*
12 hours in the ICU	28.61 ± 3.77	29.29 ± 3.58	*0*.*254*
Day of discharge	30.67 ± 3.07	31.28 ± 2.58	*0*.*193*
**Fluid balance (ml)**			
IV fluids to initiation of CPB	368.5 ± 177.0	416.4 ± 184.6	0.101
Urine to initiation of CPB	110.8 ± 95.9	135.7 ± 127.6	0.164
Fluid balance			
After 1st cardioplegia	2240.2 ± 238.8	2326.0 ± 306.4	0.055
Urine output during CPB	666.2 ± 594.0	694.0 ± 423.0	0.743
Total urine output	1326.2 ± 842.2	1419.3 ± 690.7	0.452
Use of filter, n (%)	8 (10.6%)	9 (12.0%)	0.796
Overall fluid balance	685.4 ± 784.1	809.9 ± 651.1	0.290

Transfusion data by group and comparison between the groups based on “intention to treat” are summarized in Table 4. In total, 254 units of RBCs were transfused in 150 patients. Intraoperatively, fewer group A (INVOS) patients received fewer RBC units, and the difference was statistically significant (p = 0.04). Overall, 51 of 75 patients (68%) received transfusions in the INVOS group vs. 63 of 75 patients (84%) in the control group, and this difference was highly significant (p = 0.021). Hematocrit indication was present in 20 (26.6%) group A patients (all during CPB). Transfusion *criterion a* (mean INVOS value less than 60%) was present in 37 patients and *criterion b* (INVOS ≥20% decrease) in 27 patients. Among these patients, both criteria were present in 20 patients, not necessarily the same as those showing hematocrit indication for transfusion. In total, 14 patients in this group were transfused receiving 18 units of RBCs (Table 4). Hematocrit indication was present in 21 (28.0%) patients in group B, and the proportion of patients who met the hematocrit indication for transfusion did not differ significantly between the two groups (p = 0.854). These patients were transfused along with 4 more (*see below*: *protocol violations*) receiving in total 40 units of RBCs.

**Table 4:** Transfusion data by group (analysis based on “intention to treat”)

	**Group A (n = 75)**	**Group B (n = 75)**	**P**
**In OR**			
RBC units transfused	18	40	
Patients transfused	14 (18.6%)	25 (33.3%)	*0*.*040*
RBC per transfused pt	1.29 ± 0.47	1.60 ± 0.58	*0*.*090*
RBC/pt overall	0.24 ± 0.54	0.53 ± 0.84	*0*.*011*
**In the ICU and ward**			
RBC units transfused	98	98	
Patients transfused	47 (62.6%)	57 (76.0%)	*0*.*076*
RBC per transfused pt	2.09 ± 2.05	1.71 ± 1.01	*0*.*239*
RBC/pt overall	1.31 ± 1.91	1.31 ± 1.20	*1*.*000*
**Total**			
RBC units transfused	116	138	
Patients transfused	51 (68.0%)	63 (84.0%)	*0*.*021*
RBC per transfused pt	2.27 ± 2.01	2.19 ± 1.17	*0*.*781*
RBC/pt overall	1.55 ± 1.97	1.84 ± 1.41	*0*.*288*

There were no OR deaths in either group. Duration of mechanical ventilation, ICU length of stay (LOS) and hospital LOS did not differ significantly between groups (Table 1). Fifty-one patients (68.0%) in group A and 41 patients (54.7%) in group B stayed in the hospital for more than 9 days, but most of these patients did not exhibit any pre-defined complication. In group A complications included: a) one patient for MVR/CABG = significant postoperative hemorrhage and valve dysfunction, emergency re-operation, transfusion of 15 units of RBCs, death in ICU in the 3rd day, b) ventilation time >24 h in 9 patients (3 patients were re-intubated for low cardiac output syndrome, 3 patients showed difficult and delayed weaning from mechanical ventilation, 1 patient required dialysis, 1 patient developed neurologic deficit delaying extubation but with good course, 1 patient was re-intubated for cardiac arrest during removal of thoracic drains and was re-explored), c) MI in 2 patients, and d) arrhythmias requiring treatment in 3 patients. In group B complications were: a) one patient was re-intubated for arrhythmia, hemodynamic instability, increase of hepatic enzymes and finally he died, b) ventilation time >24 h in 6 patients (1 patient was re-intubated for pneumothorax and low cardiac output syndrome, 1 patient for MI and low cardiac output syndrome, 1 patient for re-exploration for bleeding, 3 patients showed difficult and delayed weaning from mechanical ventilation), c) MI in 2 patients, d) arrhythmia requiring treatment in 2 patients, e) hemodynamic instability with cognitive dysfunction in 1 patient and f) treatment for hypertension delaying discharge in 1 patient. The overall mortality in this study was 2 deaths in 150 patients.

In total, protocol violations were identified for 13 patients (8.7%). Five of them were noted in group A (6.6%): a) one patient received 15 units of RBCs and finally died, b) two patients undergoing CABG received transfusions even though they did not meet criteria for transfusion according to our protocol, c) one patient undergoing CABG was intraoperatively transfused after meeting all (INVOS and hematocrit) criteria, but received parenteral fluids to a positive balance of 2550 ml, c) one patient undergoing CABG met criteria for transfusion but fluid balance positive by over 1.5 L. The protocol was violated in 8 group B patients (10.6%): a) one patient undergoing CABG was transfused under hematocrit indication but had postoperative fluid balance > +1.5 L; b) three patients undergoing CABG and one patient undergoing MVR were transfused without meeting the hematocrit indication (and all of them with positive fluid balance > 1.5 L) and c) three patients (one undergoing AVR/CABG and two undergoing CABG) did not receive transfusions, but had positive fluid balance >1.5 L.

After excluding cases of protocol violation, statistical analysis “per protocol” showed that, compared to the control group, significantly fewer group A patients received any blood transfusion, both intraoperatively (11 of 70 patients in group A vs. 20 of 67 patients in the control group, p = 0.048) and during the entire hospitalization (46 of 70 patients in group A vs. 55 of 67 patients in the control group, p = 0.029). Similarly, compared to the control group, the total number of RBCs transfused per patient was significantly lower in the INVOS group intraoperatively (0.20 ± 0.50 units per patient in the INVOS group vs. 0.52 ± 0.88 units per patient in the control group, p = 0.008), and during the entire hospitalization (1.31 ± 1.20 units per patient in the INVOS group vs. 1.82 ± 1.46 units per patient in the control group, p = 0.024). Of note, because the number of patients transfused and the number of RBC units transfused per patient did not differ between groups during ICU stay, the overall lower RBC use in the INVOS group seems to be the result of reduced intraoperative RBC use, while the INVOS-based protocol was in effect. Results of a per protocol analysis of our transfusion data (after excluding cases of protocol violation) are presented in Table 5.

**Table 5:** Transfusion data (“per protocol” analysis, protocol violations excluded)

	**Group A (n = 70)**	**Group B (n = 67)**	**P**
**In OR**			
RBC units transfused	14	35	
Patients transfused	11 (15.7%)	20 (29.8%)	*0*.*048*
RBC per transfused pt	1.27 ± 0.47	1.75 ± 0.55	*0*.*021*
RBC/pt overall	0.20 ± 0.50	0.52 ± 0.88	*0*.*008*
**In the ICU and ward**			
RBC units transfused	78	87	
Patients transfused	42 (60.0%)	49 (74.2%)	*0*.*103*
RBC per transfused pt	1.86 ± 0.72	1.78 ± 1.04	*0*.*670*
RBC/pt overall	1.11 ± 1.07	1.30 ± 1.25	*0*.*344*
**Overall**			
RBC units transfused	92	122	
Patients transfused	46 (65.7%)	55 (82.1%)	*0*.*029*
RBC per transfused pt	2.00 ± 0.89	2.22 ± 1.21	*0*.*314*
RBC/pt overall	1.31 ± 1.20	1.82 ± 1.46	*0.024*

## Discussion

Transfusion of RBC and other blood components in common in cardiac surgery, due to several reasons, including pre-existing anemia, intraoperative hemodilution and intraoperative or postoperative blood loss, even in otherwise uncomplicated surgery. However, even though transfusions are clearly necessary in many cases, blood product use has been associated with increased morbidity [4] and long-term mortality [3] in cardiac surgery, and therefore attempts to reduce blood transfusions may contribute to reduced complications and improved outcomes. Consequently, several measures have been evaluated in an attempt to reduce blood transfusions during cardiac surgery [12], including tolerating lower hemoglobin values [5] and restricting parenteral fluids in order to avoid hemodilution [16]. Studies evaluating the effectiveness of restrictive fluid administration strategies in mitigating the precipitous intraoperative hematocrit drop, rather than restrictive blood transfusion strategies, are scarce. We recently reported that, compared to liberal fluid administration, a restrictive parenteral fluid protocol significantly reduced intraoperative red blood cell (RBC) transfusions [17]. We also showed in a later RCT that restrictive fluid strategies may be more beneficial in patients prone to transfusion because of low preoperative hematocrit, female sex, or small BSA [16]. In both studies, re-infusion of washed blood from the thoracic cavities was used to reduce the need for transfusion. However, we faced difficulty defining a transfusion “threshold” in these studies, because existing reports raise concerns regarding safety when tolerating low hematocrit values [18,19].

Because INVOS reflects the balance of site-specific oxygen delivery vs. consumption, it is plausible to consider INVOS a reasonable monitor for adequacy of oxygen tissue delivery. Furthermore, because there is a positive correlation between INVOS and hemoglobin concentration [13], it is reasonable to interpret low INVOS values as evidence that hemoglobin or hematocrit values are not sufficient for maintaining adequate oxygen delivery to tissues, and therefore the patient needs to be transfused. Similarly, adequate INVOS values could be considered as indicator of adequate oxygen delivery, and therefore as reassurance that transfusion of red blood cells is not needed. However, data on the use of INVOS as tool for decision making with regards to blood transfusion during cardiac surgery are, to our knowledge, very limited, and we believe there is a need for rigorous assessment of this technology in cardiac surgery and perhaps in other types of major surgery.

The main finding of this prospective, randomized study was the significantly reduced intraoperative and overall need for transfusion of RBCs in patients undergoing elective cardiac surgery, with use of a hematocrit and INVOS-supported algorithm to guide the decision to transfuse, and the observed differences between the two groups were significant both in the “intention to treat”. Significant findings derived from “intention to treat” analysis are generally preferred, because results based on the “intention to treat” principle better reflect “real life” conditions, whereby errors in judgment and deviations from established protocols invariably occur. We believe that, our findings are worth reporting, because they suggest that, under optimal conditions (“per protocol analysis”) and in real-life conditions (“intention to treat” analysis), the use of INVOS to assist with decision-making regarding blood transfusions could be of value, and therefore deserves further study. In addition, we believe it is worth noting that the overall lower RBC use in group A (the INVOS group) was the result of reduced RBC use intraoperatively (while the INVOS-based protocol was in effect). Therefore, it may be reasonable to speculate that the observed overall differences between the two groups could be greater if the INVOS-protocol were applied in the ICU as well. Clearly, this is just a hypothesis, but we think this hypothesis deserves to be evaluated in a formal study.

Strengths of our study include study design (prospective, randomized, blinded), the inclusion of a well defined patient population, with clearly defined criteria for transfusion. In addition, when protocol was properly followed, reduction of RBC use was highly significant, and the observed difference between groups was clinically meaningful. Furthermore, the use of a restrictive intravenous fluid administration policy on all patients probably resulted in overall reduction of transfusion requirements in both groups, due to avoidance of hemodilution before and during CPB. Consequently, it is plausible that the observed differences between the groups, and therefore the perceived benefit from using INVOS as tool to guide transfusion decisions could be more pronounced in settings where fluid restriction is not used, and therefore overall transfusion requirements are higher.

Disadvantages of the study include the significant number of cases with protocol violations; this is probably the reason why data analysis based on “intention to treat” did now show a significant difference with regards to overall number of RBC units transfused per patient between the INVOS and the control group. Consequently, our results also demonstrate that it is difficult to conduct clinical studies in the complex operating room and ICU environment utilized for cardiac surgery, and, despite efforts to avoid them, study protocol violations can still occur. Therefore, researchers and study coordinators need to pay particular attention and closely monitor such studies in an attempt to minimize protocol violations and therefore generate more valid data.

## Conclusions

This prospective randomized clinical study suggests that the use of cerebral oximetry (INVOS) as part of an algorithm to guide RBC transfusions can result in significant reduction of RBC use in patients undergoing elective cardiac surgery, when the established protocol for fluid restriction and decision to transfuse is properly followed. However, the observed benefit is no longer significant when protocol violations are included in the analysis (“intention to treat” analysis). We suggest that, based on these results, INVOS could be a useful tool for monitoring patients during cardiac surgery, but data from well designed clinical trials with rigorous attention to study protocol, in an attempt to minimize protocol violations are needed to better assess the validity of our findings.

## Abbreviations

ACT: Activated Clotting Time; ASD: Atrial Septal Defect; AVR: Aortic Valve Replacement; BMI: Body Mass Index; BSA: Body Surface Area; CABG: Coronary Artery Bypass Grafting; COPD: Chronic Obstructive Pulmonary Disease; CPB: Cardiopulmonary Bypass; LOS: Length Of Stay; MI: Myocardial Infarction; ICU: Intensive Care Unit; INVOS: Brain Oxygen Saturation Monitoring With Near-Infrared Spectroscopy; MVR: Mitral Valve Replacement; RBC: Red Blood Cell; RCT: Randomized Controlled Trial.

## Competing interests

This research project was supported solely by department funds. All authors declare they have no conflict of interest to report.

## Authors’ contributions

GV: study design, patient care, statistical analysis, manuscript preparation, SG: patient care, data collection, KS patient care, data collection, VT patient care, data collection, DM patient care, data collection, AG study design, NT patient care, MK statistical analysis, manuscript revision, manuscript submission and correspondence. All authors have read and approved the final version submitted for publication.
